# Commercial Serological Antibody Detection Tests for the Diagnosis of Pulmonary Tuberculosis: A Systematic Review

**DOI:** 10.1371/journal.pmed.0040202

**Published:** 2007-06-12

**Authors:** Karen R Steingart, Megan Henry, Suman Laal, Philip C Hopewell, Andrew Ramsay, Dick Menzies, Jane Cunningham, Karin Weldingh, Madhukar Pai

**Affiliations:** 1 Division of Pulmonary and Critical Care Medicine, San Francisco General Hospital, University of California, San Francisco, California, United States of America; 2 Francis J. Curry National Tuberculosis Center, San Francisco, California, United States of America; 3 County of Sacramento Department of Health and Human Services, Sacramento, California, United States of America; 4 Department of Pathology, New York, New York, United States of America; 5 Department of Microbiology, New York University School of Medicine, New York, New York, United States of America; 6 Veterans Affairs Medical Center, New York, United States of America; 7 UNICEF/UNDP/World Bank/WHO Special Programme for Research and Training in Tropical Diseases, World Health Organization, Geneva, Switzerland; 8 Department of Epidemiology, Biostatistics and Occupational Health, McGill University, Montréal, Canada; 9 Respiratory Epidemiology and Clinical Research Unit, Montréal Chest Institute, Montréal, Canada; 10 Statens Serum Institut, Department of Infectious Disease Immunology, Copenhagen, Denmark; Liverpool School of Tropical Medicine, United Kingdom

## Abstract

**Background:**

The global tuberculosis epidemic results in nearly 2 million deaths and 9 million new cases of the disease a year. The vast majority of tuberculosis patients live in developing countries, where the diagnosis of tuberculosis relies on the identification of acid-fast bacilli on unprocessed sputum smears using conventional light microscopy. Microscopy has high specificity in tuberculosis-endemic countries, but modest sensitivity which varies among laboratories (range 20% to 80%). Moreover, the sensitivity is poor for paucibacillary disease (e.g., pediatric and HIV-associated tuberculosis). Thus, the development of rapid and accurate new diagnostic tools is imperative. Immune-based tests are potentially suitable for use in low-income countries as some test formats can be performed at the point of care without laboratory equipment. Currently, dozens of distinct commercial antibody detection tests are sold in developing countries. The question is “do they work?”

**Methods and Findings:**

We conducted a systematic review to assess the accuracy of commercial antibody detection tests for the diagnosis of pulmonary tuberculosis. Studies from all countries using culture and/or microscopy smear for confirmation of pulmonary tuberculosis were eligible. Studies with fewer than 50 participants (25 patients and 25 control participants) were excluded. In a comprehensive search, we identified 68 studies. The results demonstrate that (1) overall, commercial tests vary widely in performance; (2) sensitivity is higher in smear-positive than smear-negative samples; (3) in studies of smear-positive patients, Anda-TB IgG by enzyme-linked immunosorbent assay shows limited sensitivity (range 63% to 85%) and inconsistent specificity (range 73% to 100%); (4) specificity is higher in healthy volunteers than in patients in whom tuberculosis disease is initially suspected and subsequently ruled out; and (5) there are insufficient data to determine the accuracy of most commercial tests in smear microscopy–negative patients, as well as their performance in children or persons with HIV infection.

**Conclusions:**

None of the commercial tests evaluated perform well enough to replace sputum smear microscopy. Thus, these tests have little or no role in the diagnosis of pulmonary tuberculosis. Lack of methodological rigor in these studies was identified as a concern. It will be important to review the basic science literature evaluating serological tests for the diagnosis of pulmonary tuberculosis to determine whether useful antigens have been described but their potential has not been fully exploited. Activities leading to the discovery of new antigens with immunodiagnostic potential need to be intensified.

## Abstract 2

Based on a systematic review, Madhukar Pai and colleagues conclude that none of the commercial immune-based tests for pulmonary tuberculosis so far evaluated perform well enough to replace sputum smear microscopy.

## Editors' Summary

**Background.:**

Tuberculosis (TB) is, globally, one of the most important infectious diseases. It is thought that in 2005 around 1.6 million people died as a result of TB. Controlling TB requires that the disease is correctly diagnosed so that it can then be promptly treated, which will reduce the risk of infection being passed on to other individuals. The method normally used for diagnosing TB disease in poor countries (where most people with TB disease live) involves taking a sample of mucus coughed up from the lungs; this mucus is then spread thinly onto a glass slide, dyed, and viewed under the microscope. The bacteria responsible for TB take up the dye in a particular pattern and can be clearly seen under the microscope. Although this test (sputum smear) is relatively straightforward to carry out even where facilities are basic, it is not particularly good at identifying TB disease in children or amongst individuals who are HIV-positive. Finally, the sputum smear test is also not very sensitive; that is, many people who have TB disease may not give a positive reading. Therefore, there is an urgent need to develop and evaluate new tests that are suitable for use in poor countries, which will accurately diagnose TB disease, especially amongst children and people who are HIV-positive.

**Why Was This Study Done?:**

New tests for TB have become available which detect whether an individual has raised antibodies against particular proteins and other substances present on the surface of the TB bacterium. These tests are carried out on blood samples, once blood cells and other factors have been taken out. These antibody tests are often quite simple to carry out, so in principle they could be suitable for use in developing countries. Since the tests are available on the market and can be freely used in some developing countries without any need for government regulatory bodies to approve them, it is important to know how good these tests are at diagnosing TB disease. The researchers here wanted, therefore, to evaluate all of the available data relating to the accuracy of antibody detection tests for diagnosis of TB disease.

**What Did the Researchers Do and Find?:**

In order to evaluate all of the information available on commercial antibody detection tests for diagnosis of TB disease of the lungs, the researchers carried out a systematic review. First, they searched biomedical literature databases using specific terms to identify studies for inclusion. A study was included in their analysis if the commercial test was compared against one of two other standard tests (sputum smear microscopy, or growth of TB bacteria in culture). One researcher from the team then pulled out specific pieces of information from each published study: these included the type of study design; information on study participants; the type of test; what the test was compared against; and finally the results of evaluation of the test. A second researcher pulled out pieces of information from several of the same studies. The researchers then compared the information to ensure that it was recorded correctly. Each study was also assigned a quality rating, based on four distinct criteria. For each type of test, the researchers used the data in the published studies to work out the test's accuracy, both in terms of its ability to give a positive reading for people who have TB disease as well as its ability to give a negative reading for people who do not have TB disease.

The researchers found 27 papers meeting their criteria. These papers reported the results of 68 original studies. Nine different commercial tests were examined in the studies. Overall, the studies seemed to be of relatively poor quality, with only 25% of them meeting all four of the researchers' criteria for a good-quality study. The different studies appeared to produce varying results for the accuracy of these commercial tests. In particular, the tests seemed to be less accurate at detecting TB disease amongst people who had a negative sputum smear than amongst people with a positive sputum smear. When all the data for these different studies were combined, the statistics indicated that the commercial tests, overall, were only modestly accurate for diagnosis of TB disease. None of the studies had been carried out in children or in HIV-positive people.

**What Do These Findings Mean?:**

The results of this systematic review suggest that the commercial antibody detection tests considered here are not particularly useful in diagnosis of TB disease as compared to other tests, such as sputum smear and bacterial culture. Some people are concerned that there is pressure in certain developing countries to start using these tests, but the current data do not support greater use. This systematic review also highlights the fact that many studies evaluating commercial TB tests are of poor quality, and that further research needs to be done to evaluate the accuracy of different TB tests amongst children and HIV-positive patients.

**Additional Information.:**

Please access these Web sites via the online version of this summary at http://dx.doi.org/10.1371/journal.pmed.0040202.

- World Health Organization Stop TB Department website. Information about the current Stop TB strategy, data and factsheets about TB, and other resources are available
- Questions and Answers about Tuberculosis provided by the US Centers for Disease Control and Prevention
- Information about TB tests from Médicins sans Frontières (MSF). Links to MSF reports on new diagnostic tests are also available
- Wikipedia entry on Systematic Reviews (Note: Wikipedia is an internet encyclopedia anyone can edit)

## Introduction

The burden of disability and death due to tuberculosis (TB) is immense, with 8.8 million new cases of the disease and 1.6 million deaths estimated to have occurred in 2005 alone [1]. Although the incidence of TB is constant or falling in many regions of the world, rates remain high in sub-Saharan Africa as a consequence of the HIV epidemic [1,2]. The expansion of DOTS, the centerpiece of the international TB partnership control strategy, has resulted in improved case-detection rates during the past several years; however, the majority of DOTS programs in high-burden countries have fallen short of the 2005 global target of 70% case detection of the most infectious cases [1].

The vast majority of TB patients live in low- and middle-income countries [2], where the diagnosis of TB disease relies primarily on identification of acid-fast bacilli on unprocessed sputum smears using a conventional light microscope. Microscopy is highly specific for Mycobacterium tuberculosis in TB-endemic countries [3,4]. Although microscopy has been reported to have greater than 80% sensitivity for identifying cases of pulmonary TB in some settings [5,6], the sensitivity of the test has been lower and variable in other reports (range 20% to 80%) [4,7]. Moreover, sensitivity is poor for paucibacillary disease (e.g., pediatric and HIV-associated TB) [8,9], a major concern on account of the strong association between HIV infection and smear negativity [10,11]. This lack of sensitivity of the sole diagnostic test in many parts of the world results in delays in diagnosis, enabling the disease to progress and increasing the potential for transmission of M. tuberculosis [5]. To ensure appropriate care for patients and to improve control of the global TB epidemic, simple, accurate, inexpensive, and, ideally, point-of-care diagnostic tools for TB are urgently needed.

The relative importance of the different characteristics of a diagnostic test depends upon the setting in which the test is to be performed and the intended use of the results. Technical simplicity, for example, is essential if a test is to be used in a primary health-care clinic or basic health laboratory in low-income countries. If test results are to be used to exclude a diagnosis of TB in patients with respiratory symptoms in TB-endemic countries, then tests with a high sensitivity (high negative predictive value) are required even if the test is only moderately specific. Excluding TB patients from this group would then allow a more rigorous diagnostic work-up to be performed on a smaller group of patients. On the other hand, if a test is to be used to identify patients with respiratory symptoms in endemic countries for anti-TB treatment, a high specificity (high positive predictive value) is required. In the latter case, high sensitivity would also be desirable.

Immune-based tests would seem to offer the potential to improve case detection as currently performed, as some of the test formats (e.g., immunochromatographic test) are suitable for resource-limited areas. The major advantages of immune-based tests are their speed (results may be available within hours) and simplicity compared with microscopy [8,12]. The development of immune-based tests for the detection of antibodies, antigens, and immune complexes has been attempted for decades, and their performance has been critically appraised in several descriptive reviews and textbook chapters [13–22]. The most common of these tests rely on detection of the humoral (serological) antibody immune response to M. tuberculosis (the subject of this systematic review), as opposed to the T cell–based cellular immune response (e.g., interferon-gamma release assays), or direct detection of antigens in specimens other than serum (e.g., lipoarabinomannan [LAM] detection in urine [23,24] and pleural fluid [25]).

A number of in-house antibody detection tests have been developed but are not marketed. These tests use different antigens and distinct protocols and techniques.

Currently, in developing countries, where diagnostic tests are rarely subjected to regulatory review or approval [26,27], test manufacturers and distributors are marketing dozens of different antibody detection diagnostic commercial kits. The extent of their use is largely unknown; however, companies report sales volumes between 3,000 and 300,000 tests per year [28]. These tests differ in a number of their features, including antigen composition, antigen source (e.g., native or recombinant), chemical composition (e.g., protein, carbohydrate, or lipid), extent and manner of purification of the antigen(s), and class of immunoglobulin detected (e.g., IgG, IgM, or IgA). Performance data are often limited to those found in the package inserts and, being based on small sets of patients, are typically favorable (J. Cunningham, personal communication).

An antibody detection test can be developed into a number of formats depending on the membrane, antigen(s) coating, and incubation technique. Common designs include the enzyme-linked immunosorbent assay (ELISA) format and the immunochromatographic test format. ELISA is a complex assay with several steps: coating of antigens onto the surface of plastic wells, the addition of serum samples to the wells, and several washing stages. Antigen–antibody reactions are visualized using anti-human antibody linked to an enzymatic indicator system [29]. The assay can take hours to perform. The immunochromatographic test is simpler. In this technique, the antigens are pre-coated in lines across a membrane (e.g., nitrocellulose) to which samples are applied. Antigen–antibody reactions are visualized on the lines using anti-human antibody bound to substances such as colloidal gold. The test takes only a few minutes to perform [30].

The ELISA format has the advantages that many serum samples can be tested in parallel and the process can be completely automated, making this technique attractive in fully equipped laboratories that test a large number of samples. For developing countries with limited laboratory resources and access, an immunochromatographic test would be the preferred method, as this format requires only visual inspection of the antigen-containing lines and can, therefore, be performed at the point of care without laboratory equipment.

An initial survey of the literature found more than 200 studies that have evaluated commercial serological antibody detection tests, hereafter referred to as commercial tests, for the diagnosis of pulmonary TB. To our knowledge, this vast body of literature has not been systematically reviewed and synthesized. We therefore conducted a systematic review to summarize the evidence on accuracy (sensitivity and specificity) of commercial tests, according to the guidelines and methods proposed for diagnostic systematic reviews and meta-analyses [31]. We specifically addressed two questions. (1) How accurate are commercial tests for the diagnosis of pulmonary TB overall and for smear-positive and smear-negative disease? (2) What is the specificity of commercial tests in healthy control participants compared with the specificity in patients without TB, but in whom TB was initially suspected?

## Methods

### Search Strategy

We searched electronic databases for primary studies: PubMed (http://www.ncbi.nlm.nih.gov/entrez/query.fcgi?DB=pubmed) (1990 to May 2006), BIOSIS (http://scientific.thomson.com/biosis/) (1990 to October 2005), Embase (http://www.embase.com) (1990 to October 2005), and Web of Science (http://scientific.thomson.com/products/wos/) (1990 to October 2005). The search terms used included the following: “tuberculosis”, *“Mycobacterium tuberculosis”,* “immunological tests”, “serological tests”, “antibody detection”, “antigen detection”, “ELISA”, “Western blot”, and “sensitivity and specificity”. We identified additional studies by contacting experts in the field and by searching reference lists from primary studies, review articles, and textbook chapters.

### Study Selection

Our search strategy sought to identify all available articles published in English that evaluated commercial tests for the serological diagnosis of pulmonary TB. We included only those studies in which patients had bacteriologically confirmed pulmonary TB, and in which results were provided separately for smear-positive and smear-negative patients. In particular, we defined the reference standard as either isolation of M. tuberculosis on culture, or, for studies conducted without culture in endemic countries, the presence of acid-fast bacilli detected by sputum smear microscopy. No restrictions were made with respect to study design (cross sectional or case control) or data collection (prospective or retrospective).

We excluded studies that relied solely on clinical or radiological features or improvement while on anti-TB therapy as the criteria for establishing the diagnosis of TB. In addition, the following studies were excluded: (1) studies published before 1990, for the reason that many studies used crude antigen extracts or obsolete immunological methods; (2) studies with fewer than 50 participants (at least 25 TB patients and 25 control participants were required for inclusion); (3) studies of latent TB infection; (4) studies of nontuberculous mycobacteria; (5) studies of antibody responses during or after TB treatment; (6) investigations conducted using non-immunologic methods for detection of antibodies; (7) basic science literature that focuses on cloning of new antigens or their immunologic properties (e.g., epitope mapping) or other new methods of antibody detection; and (8) case reports and reviews.

Initially, two reviewers (KRS and MH) screened citations retrieved from all sources. To identify relevant studies pertaining specifically to commercial tests, a second screening was done (KRS and MH) of full texts from citations found to be relevant in the first screen. A list of excluded studies, along with the reasons for exclusion, is available from the authors on request.

### Data Extraction

We created and piloted a data-extraction form with a subset of eligible studies. Based upon experience gained in the pilot, the data-extraction form was finalized. One reviewer (KRS) extracted data from all eligible studies on the following qualities: study design and methodological quality (see assessment of study quality below), study population, antigen and antibody characteristics, laboratory technique, reference standard, and outcome data (sensitivity and specificity). To verify reproducibility of data extraction, a second reviewer (M. Henry) independently extracted data from 15% of the included studies. The inter-rater agreement between the two reviewers for sensitivity and specificity estimates was 100%. When data were not clearly reported, the information was coded as “not reported”. When necessary, we attempted to contact authors for additional information.

Although some authors compared performance of commercial tests in several different groups without TB, we preferentially selected only one comparison group (control participants) for each study in the following order: (1) patients in whom pulmonary TB was initially suspected but who were later found to have nontuberculous respiratory disease; (2) patients diagnosed with a variety of diseases other than TB (mixed disease); (3) healthy persons from endemic countries; (4) contacts of patients with TB; (5) mixed groups from categories (1) to (4); and (6) healthy persons from non-endemic countries. In our view, this hierarchy gave priority to the populations expected to be encountered in a routine clinical setting.

### Assessment of Study Quality

We assessed the quality of studies using the following criteria, which have been suggested as being important for diagnostic studies [31]. (1) Was there a comparison of the commercial test with an independent, appropriate reference standard (i.e., the commercial test did not form part of the reference standard)? (2) Was the commercial test result performed and recorded by technicians who were unaware (blinded) of the results of the reference standard? (3) Did the whole sample or a randomly selected subset of the sample receive verification using the reference standard? (4) Did the study prospectively recruit consecutive patients suspected of having pulmonary TB?

### Data Collation and Meta-Analysis

We used standard methods recommended for meta-analyses of diagnostic test evaluations [31,32]. As studies were heterogeneous, particularly with respect to the antigen composition of the tests, antibody class (IgG, IgM, or IgA), comparison groups, and sputum status of the patients, we first grouped studies by type of commercial test and then further stratified by immunoglobulin class and smear status. To calculate sensitivity and specificity values for the commercial tests, we cross-tabulated each result against the reference standard. Sensitivity refers to the proportion of pulmonary TB patients with a positive result on a given commercial test; specificity refers to the proportion of TB-negative participants who had negative results on a given commercial test. Whenever possible, we extracted raw data from primary studies to fill the four cell values of a diagnostic 2 × 2 table: true positives, false positives, true negatives, and false negatives. In calculations of sensitivity, we included studies from endemic countries that used sputum smear positivity as the reference standard along with studies using culture as the reference standard. We recognized that some authors used the same comparison group for multiple studies, and thus derived identical specificity estimates. Therefore, in determining specificity, we included the specificity value for the specific comparison group only once when appropriate. For clarity of presentation, studies that reported results stratified by subgroups are shown more than once in tables or figures.

Data were analyzed using SPSS (version 14.0.1.366) [33] and Meta-DiSc (version 1.4) software [34]. Sensitivity and specificity values were calculated for the commercial tests investigated in each study, along with their 95% confidence intervals (CIs). In addition to the sensitivity and specificity estimates and forest plots generated for this review, true positive rates (TPR = sensitivity) and false positive rates (FPR = 1 − specificity) were summarized using an asymmetric summary receiver operating characteristic (SROC) curve [35]. TPR and FPR are not independent of each other as they vary with the thresholds (cut points for determining test positives) employed in the original studies. In addition, it is likely that different thresholds were used in various studies, either implicitly or explicitly. Because of the inherent trade-off between TPR and FPR, it is imperative to plot the estimates of the two quantities in a receiver operating characteristic (ROC) space and to use meta-analytic methods that take into account the threshold effect. Thus, we did not pool the sensitivity and specificity estimates separately; instead we analyzed TPR and FPR as pairs in an SROC analysis, and explored the effect of variability in cut points on study results.

Unlike a traditional ROC plot that explores the effect of varying thresholds on sensitivity and specificity in a single study, each data point in the SROC space represents an individual study. As described by Littenberg and Moses [32], the SROC analysis involves three steps: (1) the pairs of TPR and FPR estimates from each study are transformed onto a suitable scale of log odds; (2) a linear regression equation is fitted using the transformed data; and (3) the coefficients from the linear regression model are used to generate a curve in the original ROC space.

The area under the curve (AUC) (in this case, being the area under the SROC curve) presents an overall summary of test performance and displays the trade-off between sensitivity and specificity. An AUC of 1.0 (100%) indicates perfect discriminatory ability of the diagnostic test. In addition, the Q* index is another useful global estimate of test accuracy for comparing SROC curves. The Q* index, defined by the point where sensitivity equals specificity on the SROC curve, is the point on the SROC curve that is intersected by the anti-diagonal. A Q* value of 1.0 indicates 100% accuracy (sensitivity and specificity of 100%) [32,35,36].

In meta-analyses of studies of diagnostic tests, heterogeneity refers to a high level of variability in study results [37]. Such heterogeneity could be a result of variability in thresholds, laboratory technique, disease spectrum, study design, and/or quality between studies [37]. In the presence of significant heterogeneity, pooled or summary estimates from meta-analyses are hard to interpret. Given the anticipated variability in accuracy, we decided, a priori, to avoid direct pooling of sensitivity and specificity values. Also, as described earlier, we addressed heterogeneity by using subgroup (stratified) analyses.

## Results

### Description of Included Studies

**Figure 1:**
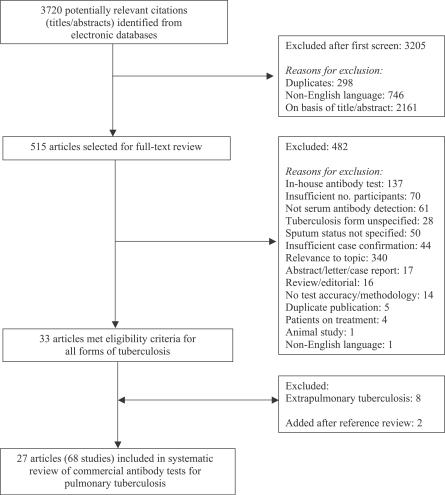
Flow Diagram for Study Selection

From the literature searches, we identified 3,720 citations, of which 27 publications (68 studies) met our eligibility criteria (Figure 1) [38–64]. We considered most studies to be distinct ([43], study b, is a substudy). Therefore, no effort was made to account for lack of independence. Of the total 68 studies, 32 (47%) collected data prospectively and 36 (53%), retrospectively. Twenty-four (35%) studies used either random or consecutive recruitment of participants, while 44 (65%) studies did not report the method of participant selection. Thirty-one (46%) studies reported at least single-blinded interpretation of commercial test and reference standard results. Mycobacterial culture was used as the gold standard in 51 (75%) studies and sputum smear microscopy was used in 17 (25%) studies. For all studies, the commercial test did not form part of the reference standard. In 39 (57%) studies, the entire study population was investigated using the identical reference standard (complete verification), while in 18 (26%) studies, the reference standard for TB patients and control participants differed (e.g., mycobacterial culture for TB patients and chest radiograph for control participants [differential verification]). For 11 (16%) studies, information about verification was unclear or not reported. Seventeen (25%) studies met all four criteria for good quality [43,48,53]. Forty-one (60%) studies were performed with smear microscopy–positive patients, and 27 (40%) were conducted with smear microscopy–negative patients. No studies involved children younger than 15 y old or patients with HIV infection. The median number of TB patients was 41 (interquartile range 38 to 75); the median number of control participants in comparison groups was 45 (interquartile range 40 to 107).

Antibody detection was done with stored, frozen sera in 59 (87%) studies and with fresh sera in one (2%) study [60]; in eight (12%) studies, the condition of the specimens was not reported. A total of nine different commercial tests are included in the review. For seven (78%) commercial tests, the specific antigen composition was described; for two (22%) tests, the antigen composition was considered a proprietary product. Fifty-nine (87%) studies assessed the performance of individual commercial tests. Nine (13%) studies evaluated the performance of two or more commercial tests used in combination. In these nine studies, the commercial tests (Pathozyme TB Complex Plus and Pathozyme Myco) came from the same manufacturer, but differed in their antigen composition and/or immunoglobulin class. Table 1 lists selected characteristics of the commercial tests employed in the review, together with the names and addresses of their respective manufacturers.

**Table 1:**
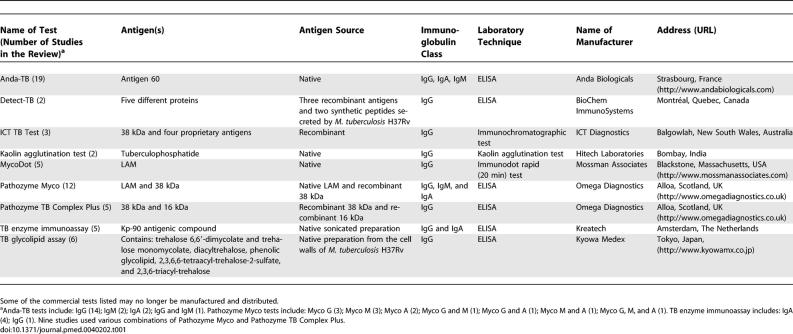
Commercial Antibody Detection Tests for the Diagnosis of Pulmonary TB

### How Accurate are Commercial Tests for the Diagnosis of Pulmonary TB Overall and for Smear-Positive and Smear-Negative Disease?

**Figure 2:**
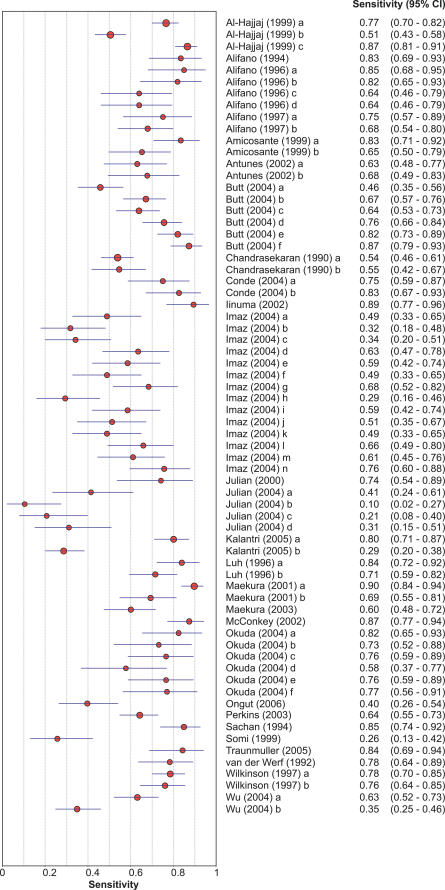
Sensitivity Estimates of Commercial Tests for the Diagnosis of Pulmonary TB The circles and lines represent the point estimates and 95% CIs, respectively. The size of the circle indicates the study size.

**Figure 3:**
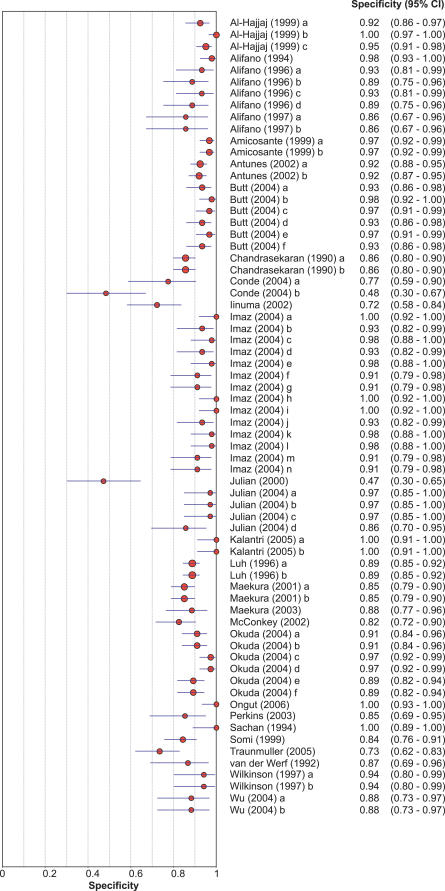
Specificity Estimates of Commercial Tests for the Diagnosis of Pulmonary TB The circles and lines represent the point estimates and 95% CIs, respectively. The size of the circle indicates the study size.

**Figure 4:**
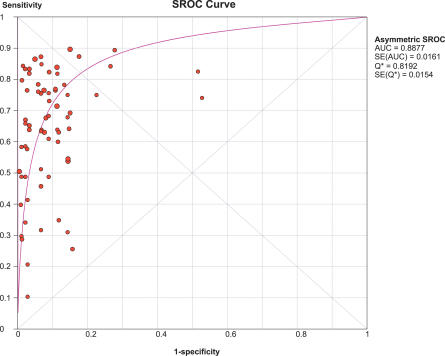
SROC Curve of Commercial Tests for the Diagnosis of Pulmonary TB Each solid circle represents an individual study in the meta-analysis. The curve is the regression line that summarizes the overall diagnostic accuracy. SE (AUC), standard error of AUC; Q*, an index defined by the point on the SROC curve where the sensitivity and specificity are equal; SE (Q*), standard error of Q* index.

Tables 2–10 show performance and other selected characteristics for the commercial tests in the review. When all 68 studies were considered together, the sensitivity estimates ranged from 10% to 90% (Figure 2), and the specificity estimates ranged from 47% to 100% (Figure 3). On the whole, both measures varied widely among studies of a given commercial test and in studies across different commercial tests. Figure 4 shows performance for all commercial tests combined in a SROC curve. The AUC was 0.89 (95% CI 0.86–0.92), and the Q* index was 0.82, indicating modest accuracy.

**Table 2:**
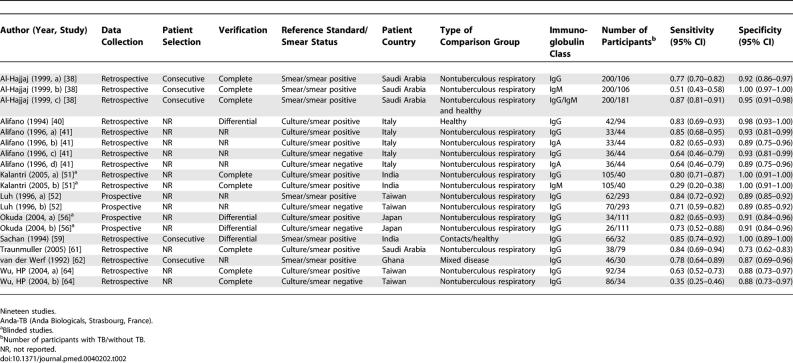
Selected Characteristics of Studies Investigating Anda-TB for the Diagnosis of Pulmonary TB

**Table 3:**
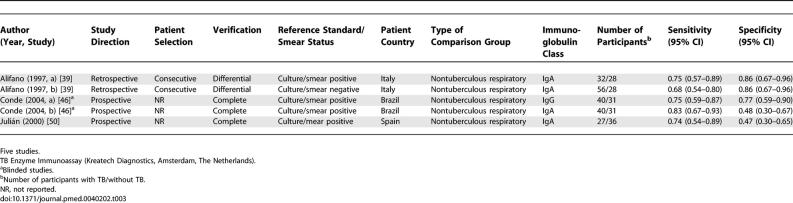
Selected Characteristics of Studies Investigating TB Enzyme Immunoassay for the Diagnosis of Pulmonary TB

**Table 4:**
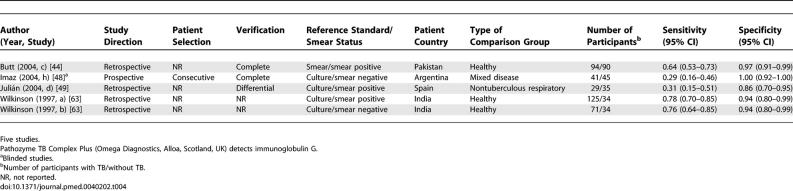
Selected Characteristics of Studies Investigating Pathozyme TB Complex Plus for the Diagnosis of Pulmonary TB

**Table 5:**
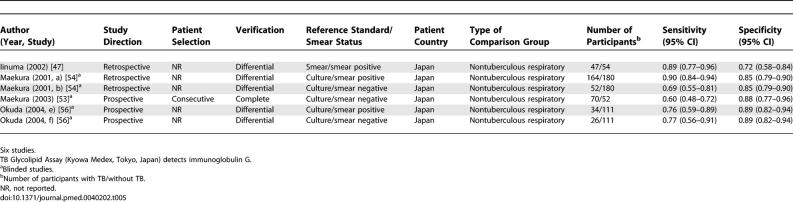
Selected Characteristics of Studies Investigating TB Glycolipid Assay for the Diagnosis of Pulmonary TB

**Table 6:**
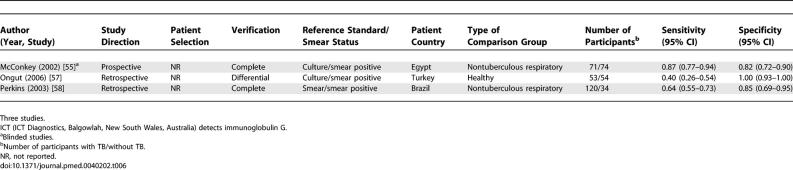
Selected Characteristics of Studies Investigating ICT for the Diagnosis of Pulmonary TB

**Table 7:**
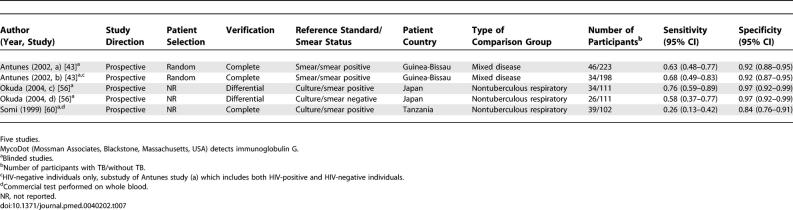
Selected Characteristics of Studies Investigating MycoDot for the Diagnosis of Pulmonary TB

**Table 8:**
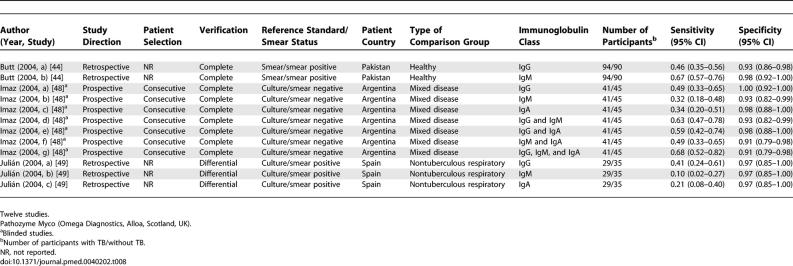
Selected Characteristics of Studies Investigating Pathozyme Myco for the Diagnosis of Pulmonary TB

**Table 9:**
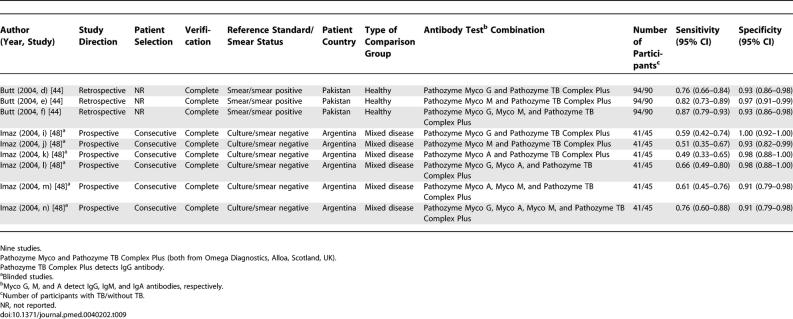
Selected Characteristics of Studies Investigating Pathozyme Myco and Pathozyme TB Complex Plus in Various Combinations for the Diagnosis of Pulmonary TB

**Table 10:**
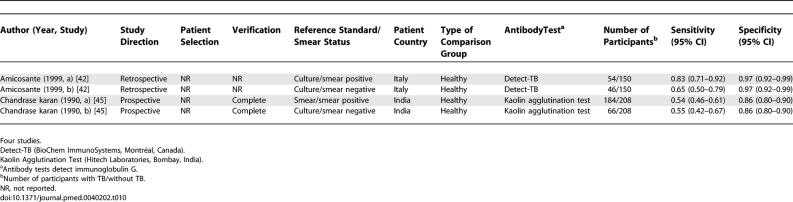
Selected Characteristics of Studies Investigating Detect-TB and Kaolin Agglutination Test for the Diagnosis of Pulmonary TB

**Figure 5:**
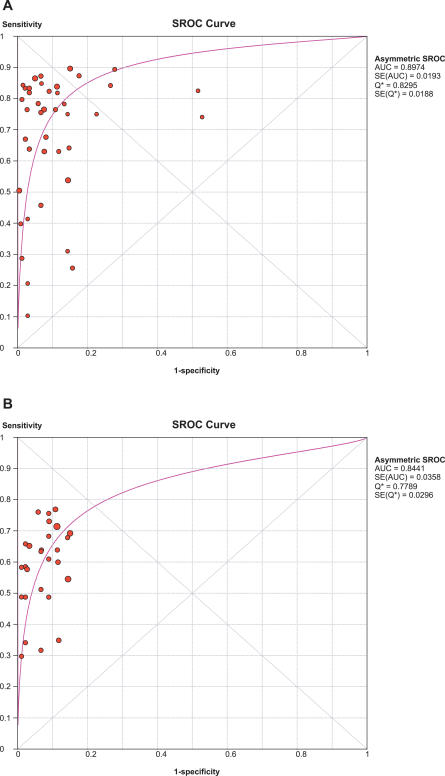
SROC Curve of Commercial Tests for the Diagnosis of Pulmonary TB (A) Smear microscopy–positive patients; (B) smear microscopy–negative patients. Each solid circle represents an individual study in the meta-analysis. The curve is the regression line that summarizes the overall diagnostic accuracy. SE (AUC), standard error of AUC; Q*, an index defined by the point on the SROC curve where the sensitivity and specificity are equal; SE (Q*), standard error of Q* index.

The accuracy of commercial tests in patients with smear-positive pulmonary TB was higher (AUC = 0.90, 95% CI 0.86–0.94; 41 studies [Figure 5A]) than in patients with smear-negative disease (AUC = 0.84, 95% CI 0.77–0.91; 27 studies [Figure 5B]). These differences were, however, not statistically significant, based on overlapping CIs for the AUCs. Corresponding forest plots of sensitivity and specificity are shown in Figures S1–S4.

**Figure 6:**
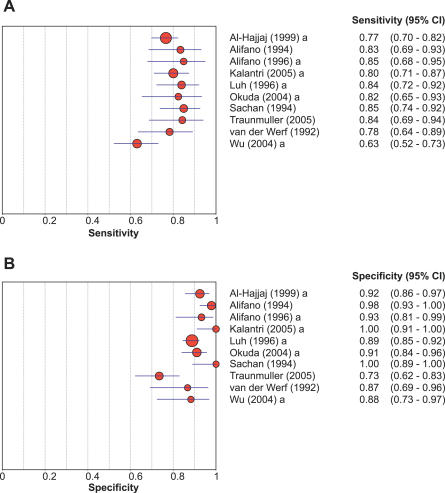
Sensitivity and Specificity Estimates of Anda-TB IgG for the Diagnosis of Pulmonary TB, Smear Microscopy–Positive Patients (A) Sensitivity; (B) specificity. The circles and lines represent the point estimates and 95% CIs, respectively. The size of the circle indicates the study size.

Anda-TB IgG was the test most frequently evaluated in the review. Figure 6 shows the forest plots for studies using Anda-TB IgG for the diagnosis of pulmonary TB in smear microscopy–positive patients (ten studies) [38,40,41,51,52,56,59,61,62,64]. Figure S5 shows the corresponding SROC curve. Sensitivity values ranged from 63% to 85%, with sensitivity ≤ 80% in four (40%) studies [38,51,62,64]; specificity values ranged from 73% to 100%, with specificity < 90% in four (40%) studies [52,61,62,64]. The AUC was 0.86 (95% CI 0.77–0.95), which was lower than the value noted in studies of smear microscopy–positive patients for all commercial tests combined. In smear microscopy–negative patients (four studies) [41,52,56,64], sensitivity estimates for Anda-TB IgG were low and variable (64%, 71%, 73%, and 35%); corresponding specificity estimates were similar (93%, 89%, 91%, and 88%) (Table 2). After stratifying by smear status, there were fewer than four studies for the other commercial tests. For each of these studies, sensitivity and specificity values are shown in Tables 2–10.

### What is the Specificity of Commercial Tests in Healthy Control Participants Compared with the Specificity in Patients without TB, but in Whom TB was Initially Suspected?

**Figure 7:**
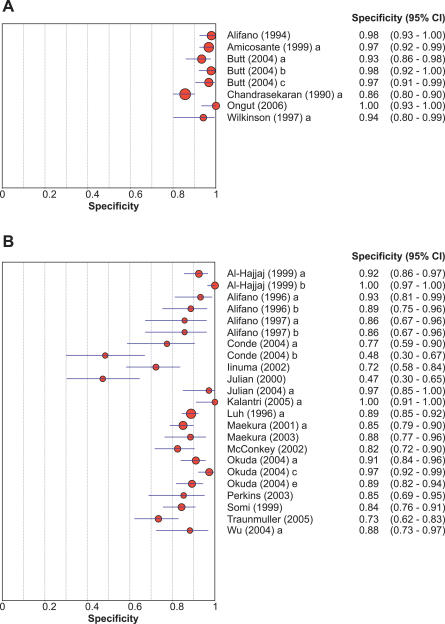
Specificity Estimates of Commercial Tests (A) Healthy control participants; (B) patients with nontuberculous respiratory disease. Studies using identical comparison groups appear only once. The circles and lines represent the point estimates and 95% CIs, respectively. The size of the circle indicates the study size. doi:10.1371/journal.pmed.0040202.g007

We determined specificity estimates for a subgroup of studies involving healthy participants [40,42,44,45,57,63] compared to patients in whom TB disease was initially considered and subsequently ruled out (nontuberculous respiratory disease) [38,39,41,46,47,49–56,58,60,61,64]. In healthy volunteers, specificity values ranged from 86% to 100%, with seven (88%) studies [40,42,44,57,63] demonstrating specificity values higher than 90%. In comparison, in studies of patients with nontuberculous respiratory disease (23 studies), specificity values ranged from 47% to 100%; specificity values were higher than 90% in only seven (30%) studies [38,41,49,51,56] (Figure 7). Compared with studies involving patients with nontuberculous respiratory disease, studies in healthy volunteers showed a higher value for the AUC (0.91 [95% CI 0.73–1.10] versus 0.87 [95% CI 0.82–0.92]), although this difference was not statistically significant, based on overlapping CIs (Figure S6A and S6B).

## Discussion

### Principal Findings

Our systematic review of 68 studies examining the performance of commercial tests for the diagnosis of pulmonary TB suggests that (1) overall, commercial tests vary widely in performance; (2) sensitivity is higher in smear-positive than smear-negative samples; (3) in studies of smear-positive patients, Anda-TB IgG by ELISA shows limited sensitivity (range 63% to 85%) and inconsistent specificity (range 73% to 100%); (4) specificity is higher in healthy volunteers than in patients in whom TB disease is initially suspected and subsequently ruled out; and (5) there are insufficient data to determine the accuracy of most commercial tests in smear-negative patients, as well as their performance in children or persons with HIV infection.

Our systematic review had several strengths. First, the comprehensive search strategy with various overlapping approaches enabled us to retrieve relevant studies published since 1990. Moreover, two reviewers independently completed screening and study selection. To verify reproducibility of data extraction, a second reviewer independently extracted data on sensitivity, specificity and study quality in 15% of the included studies. We contacted authors for missing data. None of the studies in the review used the result from the antibody test as a reference to confirm TB (incorporation bias). When possible, we selected studies using patients with disease, in preference to healthy control participants, to evaluate how well antibody tests performed in patients with and without pulmonary TB among persons suspected of having TB. Finally, we analyzed data within specific subgroups to lessen the effect of heterogeneity.

This review also had limitations. Except for Anda-TB IgG, there were not sufficient relevant studies for specific commercial tests to provide meaningful summary measures of performance, particularly in smear-negative samples. In addition, there were no studies that met our inclusion criteria for the diagnosis of pulmonary TB in children or in patients with HIV infection. Another problem concerned limited information on clinical status and disease severity in the populations tested. Differing criteria for patient selection and greater duration and severity of illness of the study populations may have introduced significant variability in findings among studies (selection bias). Moreover, different settings (e.g., areas of high HIV prevalence, hospitals, and out-patient clinics) may have accounted for some of the observed variation in test performance. The condition of specimens may have affected the sensitivity results. Only one study [60] used fresh serum; the vast majority (87%) of studies used frozen sera; thus samples were subjected to an unknown freeze–thaw cycle history, which may decrease sensitivity [58]. A majority (75%) of studies used mycobacterial culture as the reference standard. Although in TB diagnostic trials, mycobacterial culture is considered the gold standard, culture does not detect all cases of TB; therefore, some degree of misclassification of disease for study participants was possible. We included studies from endemic countries that used sputum smear microscopy, a test with modest sensitivity (up to 70%–80%), as the reference standard; use of an insensitive reference standard may have led to biased estimates of commercial test accuracy [65]. The choice of the reference standard (culture and/or smear) for this review was a problem for pediatric TB. In this group, it is difficult to diagnose pulmonary TB on the basis of bacteriologic confirmation [9].

Another set of problems involved shortcomings in study design and quality. Only 24 (35%) studies recruited participants in a random or consecutive manner. Therefore, most studies lacked the sound probabilistic sampling framework possible in consecutive or random sampling designs. Only approximately half of the studies (31 [46%]) reported blinded interpretation of the results of the commercial test and the reference standard, a major limitation of the currently available literature. Lack of blinding may have resulted in an overestimation of the sensitivity of the commercial test result [66]. In 29 (43%) studies, different diagnostic tests were performed in TB patients and control participants (mycobacterial culture for patients and chest radiographs for control participants [verification bias]), or information about the tests to rule out TB, if performed, was not reported. Only 17 (25%) studies met all four criteria for good quality. Variability in study design and study quality might account for some of the observed heterogeneity evident in the results. Although statistical tests and graphical methods are available to detect potential publication bias in meta-analyses of randomized trials, such techniques have not been adequately evaluated for diagnostic data [67]. Thus, it is difficult to rule out publication bias in our review. In addition, our search strategy may have missed some relevant studies by excluding non-English publications. This represented approximately 20% of all excluded studies.

Finally, because the antigen content of commercial tests is sometimes considered proprietary information, we could not determine the antigen composition for all tests in the review. Test manufacturers may change their product without forewarning and, in some countries, such as Brazil, products are registered in the distributor's name; a change in distributor will therefore result in a change in product name, making it difficult to ascertain which tests are in current use. These points argue for independent evaluations of new immune-based diagnostic tests and, by extension, greater regulatory oversight of diagnostic tests in general, particularly for diseases that have a significant public health impact.

Despite the long history of failed attempts to develop an accurate serodiagnostic test for TB and the inadequate performance of the current commercial tests, it is important to emphasize that studies during the past decade have not only permitted an understanding of the lacunae in the efforts so far [19,20,68,69], but have also identified several promising candidate antigens [19,20,68–73]. A systematic analysis of the humoral immune responses of TB patients has shown that the profile of antigenic proteins of M. tuberculosis recognized by antibodies differs at different stages of infection and disease progression [68,69,74,75] and thus, an accurate diagnostic test for TB will need to be based on a combination of antigens [68,69,75]. In HIV-infected TB patients, several antigens that are recognized by antibodies have been delineated, however, only the 81-kDa malate synthase protein has been evaluated in patients from different countries [19,20,70–73]. It is encouraging that this antigen provided equivalent sensitivity in patients from India, the United States, and Uganda [19,20,70] since it has been suggested that differences in genetic make-up might account for the observed variations in antibody responses to specific M. tuberculosis antigens [13,76].

Antigens that are immunodominant during extrapulmonary and pediatric TB also need to be identified. A systematic review of 21 studies evaluating the performance of commercial tests for the diagnosis of extrapulmonary TB showed highly variable estimates of sensitivity and specificity, and no studies were of sufficient quality to enable their evaluation in patients with HIV infection or in children—the two groups for which the test would be most useful [77].

### Conclusions and Policy Implications

The evidence provided in this systematic review suggests that, at this point in time, published data on commercial antibody detection tests produce inconsistent estimates of accuracy, and none of the assays perform well enough to replace sputum smear microscopy. These tests thus have little or no role to play in the diagnosis of pulmonary TB at the present time. Given these findings, we express concern that commercial tests in present use may divert resources in developing countries away from smear microscopy. Our findings underscore the need for greater regulatory oversight of in vitro diagnostics and improved capacity in countries to design, conduct, and report diagnostic test evaluations which, in turn, can guide procurement and clinical practice. Lack of methodological rigor in these studies was a cause for concern. Recent articles have attested to the mediocre quality of diagnostic studies for TB [26,78]. Use of guidelines such as the Standards for Reporting of Diagnostic Accuracy (STARD) [79] and the tool for quality assessment of diagnostic accuracy studies (the QUADAS tool) [80] may lead to improvements in the quality of future studies. Guidelines specifically for the evaluation of diagnostic tests for infectious diseases have recently been published [81].

It is important that the literature from research laboratories that have evaluated the immunodiagnostic potential of different antigens be reviewed to determine whether there are useful antigens which have been described but whose potential has not been fully exploited. The increased understanding of the humoral immune responses in TB patients and the new tools of genomics and proteomics could well lead to devising the simple, rapid, and accurate serodiagnostic test that that has eluded us so far. Activities aimed at discovering new antigens with immunodiagnostic potential need to be intensified. Trials of new serodiagnostic tests for pulmonary TB must adequately address the particular challenges presented by smear microscopy–negative patients, children, and persons with HIV infection.

## Supporting Information

Figure S1 Sensitivity Estimates of Commercial Tests for the Diagnosis of Pulmonary TB, Smear Microscopy–Positive Patients The circles and lines represent the point estimates and 95% CIs, respectively. The size of the circle indicates the study size. Numbers in parentheses indicate references. EIA, enzyme immunoassay; IgG, IgM, IgA (G, M, A), immunoglobulin G, M, A, respectively; KAT, kaolin agglutination test; P Plus, Pathozyme TB Complex Plus; Path, Pathozyme; TBGL, tuberculosis glycolipid assay.
(262 KB PDF)

Figure S2 Specificity Estimates of Commercial Tests for the Diagnosis of Pulmonary TB, Smear Microscopy–Positive Patients The circles and lines represent the point estimates and 95% CIs, respectively. The size of the circle indicates the study size. Numbers in parentheses indicate references. EIA, enzyme immunoassay; IgG, IgM, IgA (G, M, A), immunoglobulin G, M, A, respectively; KAT, kaolin agglutination test; P Plus, Pathozyme TB Complex Plus; Path, Pathozyme; TBGL, tuberculosis glycolipid assay.
(257 KB PDF)

Figure S3 Sensitivity Estimates of Commercial Tests for the Diagnosis of Pulmonary TB, Smear Microscopy–Negative Patients The circles and lines represent the point estimates and 95% CIs, respectively. The size of the circle indicates the study size. Numbers in parentheses indicate references. EIA, enzyme immunoassay; IgG, IgM, IgA (G, M, A), immunoglobulin G, M, A, respectively; KAT, kaolin agglutination test; P Plus, Pathozyme TB Complex Plus; Path, Pathozyme; TBGL, tuberculosis glycolipid assay.
(259 KB PDF)

Figure S4 Specificity Estimates of Commercial Tests for the Diagnosis of Pulmonary TB, Smear Microscopy–Negative Patients The circles and lines represent the point estimates and 95% CIs, respectively. The size of the circle indicates the study size. Numbers in parentheses indicate references. EIA, enzyme immunoassay; IgG, IgM, IgA (G, M, A), immunoglobulin G, M, A, respectively; KAT, kaolin agglutination test; P Plus, Pathozyme TB Complex Plus; Path, Pathozyme; TBGL, tuberculosis glycolipid assay.
(256 KB PDF)

Figure S5 SROC Curve of Anda-TB IgG for the Diagnosis of Pulmonary TB, Smear Microscopy–Positive Patients Each solid circle represents an individual study in the meta-analysis. The curve is the regression line that summarizes the overall diagnostic accuracy. SE (AUC), standard error of AUC; Q*, an index defined by the point on the SROC curve where the sensitivity and specificity are equal; SE (Q*), standard error of Q* index.
(234 KB PDF)

Figure S6 SROC Curve of Commercial Tests for the Diagnosis of Pulmonary TB (A) Healthy control participants; (B) patients with nontuberculous respiratory disease. Each solid circle represents an individual study in the meta-analysis. The curve is the regression line that summarizes the overall diagnostic accuracy. SE (AUC), standard error of AUC; Q*, an index defined by the point on the SROC curve where the sensitivity and specificity are equal; SE (Q*), standard error of Q* index.
(266 KB PDF)

## Abbreviations

AUC: area under the curve
CI: confidence interval
ELISA: enzyme-linked immunosorbent assay
FPR: false positive rate
LAM: lipoarabinomannan
ROC: receiver operating characteristic
SROC: summary receiver operating characteristic
TB: tuberculosis
TPR: true positive rate
