# Correction: Commercial Serological Antibody Detection Tests for the Diagnosis of Pulmonary Tuberculosis: A Systematic Review

**DOI:** 10.1371/journal.pmed.0040254

**Published:** 2007-08-28

**Authors:** Karen R Steingart, Megan Henry, Suman Laal, Philip C Hopewell, Andrew Ramsay, Dick Menzies, Jane Cunningham, Karin Weldingh, Madhukar Pai

Correction for:

Steingart KR, Henry M, Laal S, Hopewell PC, Ramsay A, et al. (2007) Commercial Serological Antibody Detection Tests for the Diagnosis of Pulmonary Tuberculosis: A Systematic Review. PLoS Med 4(6): e202. doi:10.1371/journal.pmed.0040202

Figure 1 contained an error. Of the 515 articles selected for full-text review, in the box entitled “Excluded,” the number for “Relevance to topic” should read 34, not 340. The revised figure appears below:

**Figure 1:**
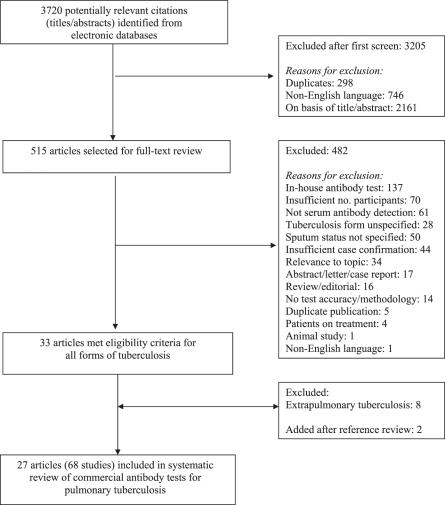
Flow Diagram for Study Selection

