# Supplementary material for: Commercial Serological Antibody Detection Tests for the Diagnosis of Pulmonary Tuberculosis: A Systematic Review
Source: PLoS Med. 2007 Jun 12;4(6):e202. doi: 10.1371/journal.pmed.0040202 (PMC1891320; doi:10.1371/journal.pmed.0040202)
Supplement: Supplementary file 1 [file pmed.0040202.sg001.pdf]

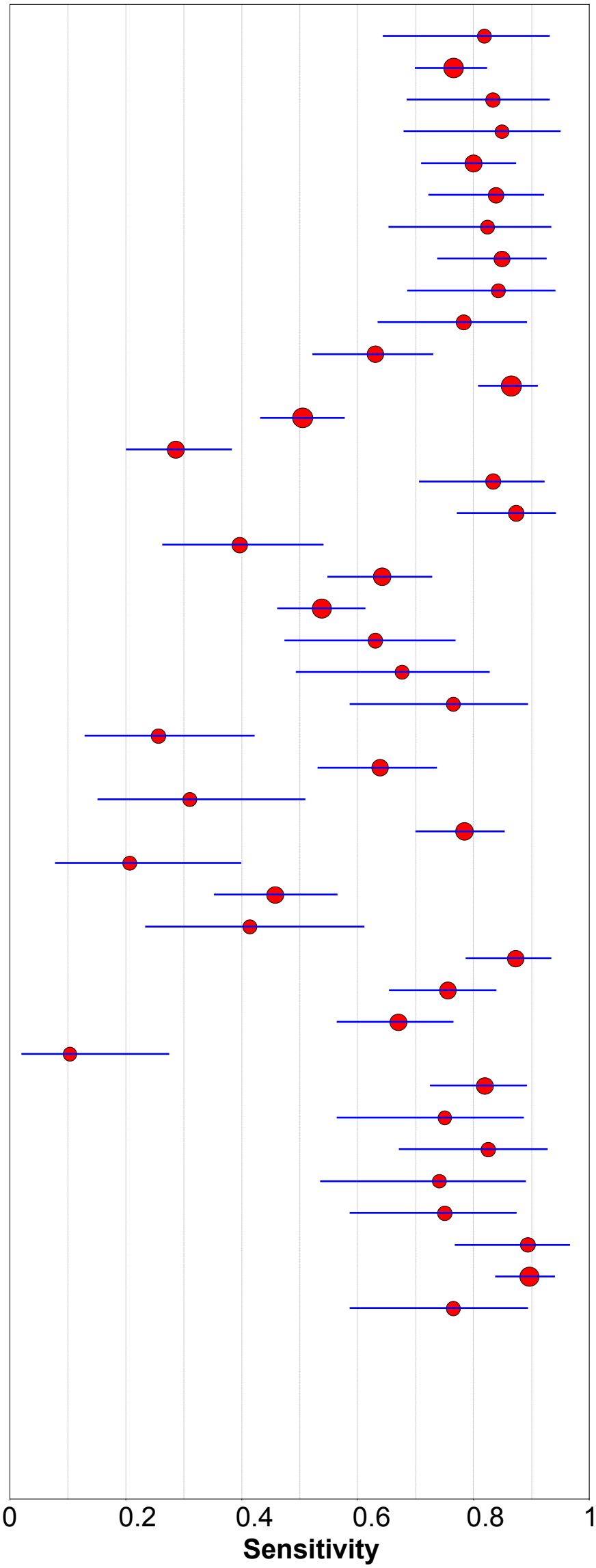

Study	Sensitivity	(95% CI)
Anda IgA (41)	0.82	(0.65 - 0.93)
Anda IgG (38)	0.77	(0.70 - 0.82)
Anda IgG (40)	0.83	(0.69 - 0.93)
Anda IgG (41)	0.85	(0.68 - 0.95)
Anda IgG (51)	0.80	(0.71 - 0.87)
Anda IgG (52)	0.84	(0.72 - 0.92)
Anda IgG (56)	0.82	(0.65 - 0.93)
Anda IgG (59)	0.85	(0.74 - 0.92)
Anda IgG (61)	0.84	(0.69 - 0.94)
Anda IgG (62)	0.78	(0.64 - 0.89)
Anda IgG (64)	0.63	(0.52 - 0.73)
Anda IgG/IgM (38)	0.87	(0.81 - 0.91)
Anda IgM (38)	0.51	(0.43 - 0.58)
Anda IgM (51)	0.29	(0.20 - 0.38)
Detect TB (42)	0.83	(0.71 - 0.92)
ICT (55)	0.87	(0.77 - 0.94)
ICT (57)	0.40	(0.26 - 0.54)
ICT (58)	0.64	(0.55 - 0.73)
KAT (45)	0.54	(0.46 - 0.61)
MycoDot (43)	0.63	(0.48 - 0.77)
MycoDot (43)	0.68	(0.49 - 0.83)
MycoDot (56)	0.76	(0.59 - 0.89)
MycoDot (60)	0.26	(0.13 - 0.42)
P Plus (44)	0.64	(0.53 - 0.73)
P Plus (49)	0.31	(0.15 - 0.51)
P Plus (63)	0.78	(0.70 - 0.85)
Path Myco A (49)	0.21	(0.08 - 0.40)
Path Myco G (44)	0.46	(0.35 - 0.56)
Path Myco G (49)	0.41	(0.24 - 0.61)
Path Myco G/M/P Plus (44)	0.87	(0.79 - 0.93)
Path Myco G/P Plus (44)	0.76	(0.66 - 0.84)
Path Myco M (44)	0.67	(0.57 - 0.76)
Path Myco M (49)	0.10	(0.02 - 0.27)
Path Myco M/P Plus (44)	0.82	(0.73 - 0.89)
TB IgA EIA (39)	0.75	(0.57 - 0.89)
TB IgA EIA (46)	0.83	(0.67 - 0.93)
TB IgA EIA (50)	0.74	(0.54 - 0.89)
TB IgG EIA (46)	0.75	(0.59 - 0.87)
TBGL (47)	0.89	(0.77 - 0.96)
TBGL (54)	0.90	(0.84 - 0.94)
TBGL (56)	0.76	(0.59 - 0.89)
