# Supplementary material for: Commercial Serological Antibody Detection Tests for the Diagnosis of Pulmonary Tuberculosis: A Systematic Review
Source: PLoS Med. 2007 Jun 12;4(6):e202. doi: 10.1371/journal.pmed.0040202 (PMC1891320; doi:10.1371/journal.pmed.0040202)
Supplement: Supplementary file 2 [file pmed.0040202.sg002.pdf]

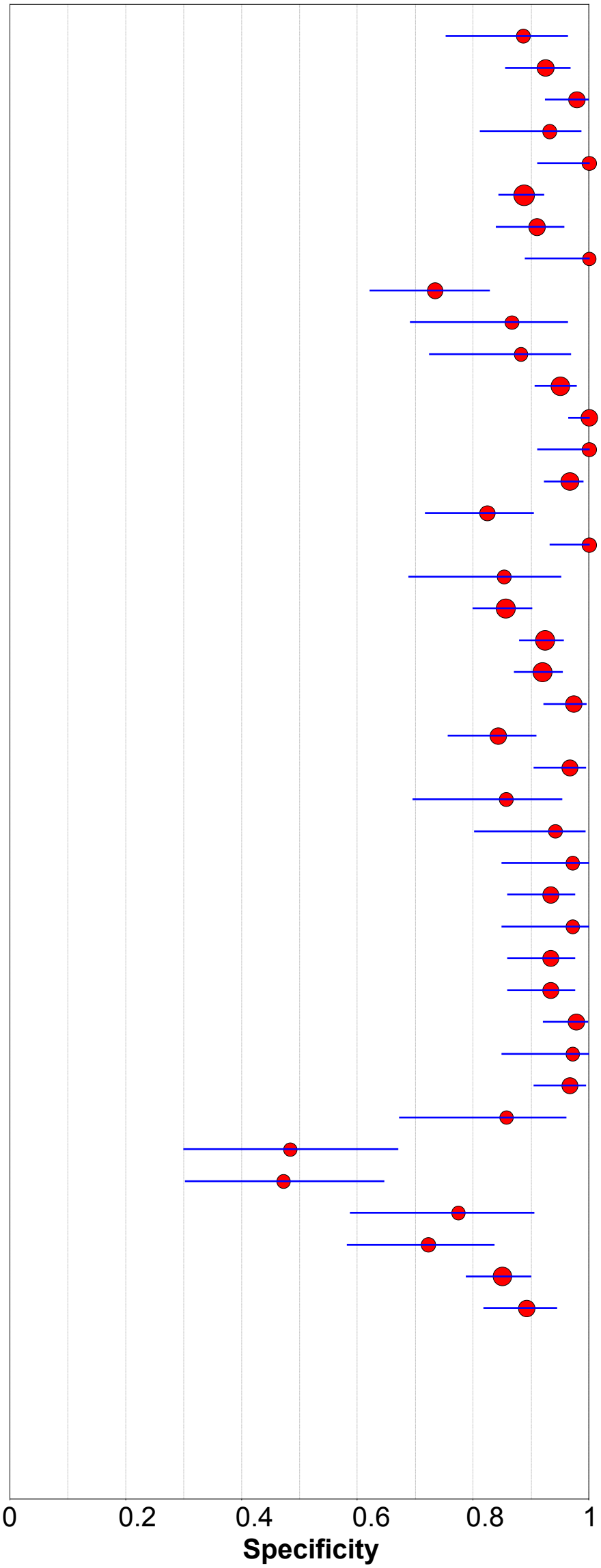

	Specificity (95% CI)	
Anda IgA (41)	0.89	(0.75 - 0.96)
Anda IgG (38)	0.92	(0.86 - 0.97)
Anda IgG (40)	0.98	(0.93 - 1.00)
Anda IgG (41)	0.93	(0.81 - 0.99)
Anda IgG (51)	1.00	(0.91 - 1.00)
Anda IgG (52)	0.89	(0.85 - 0.92)
Anda IgG (56)	0.91	(0.84 - 0.96)
Anda IgG (59)	1.00	(0.89 - 1.00)
Anda IgG (61)	0.73	(0.62 - 0.83)
Anda IgG (62)	0.87	(0.69 - 0.96)
Anda IgG (64)	0.88	(0.73 - 0.97)
Anda IgG/IgM (38)	0.95	(0.91 - 0.98)
Anda IgM (38)	1.00	(0.97 - 1.00)
Anda IgM (51)	1.00	(0.91 - 1.00)
Detect TB (42)	0.97	(0.92 - 0.99)
ICT (55)	0.82	(0.72 - 0.90)
ICT (57)	1.00	(0.93 - 1.00)
ICT (58)	0.85	(0.69 - 0.95)
KAT (45)	0.86	(0.80 - 0.90)
MycoDot (43)	0.92	(0.88 - 0.95)
MycoDot (43)	0.92	(0.87 - 0.95)
MycoDot (56)	0.97	(0.92 - 0.99)
MycoDot (60)	0.84	(0.76 - 0.91)
P Plus (44)	0.97	(0.91 - 0.99)
P Plus (49)	0.86	(0.70 - 0.95)
P Plus (63)	0.94	(0.80 - 0.99)
Path Myco A (49)	0.97	(0.85 - 1.00)
Path Myco G (44)	0.93	(0.86 - 0.98)
Path Myco G (49)	0.97	(0.85 - 1.00)
Path Myco G/M/P Plus (44)	0.93	(0.86 - 0.98)
Path Myco G/P Plus (44)	0.93	(0.86 - 0.98)
Path Myco M (44)	0.98	(0.92 - 1.00)
Path Myco M (49)	0.97	(0.85 - 1.00)
Path Myco M/P Plus (44)	0.97	(0.91 - 0.99)
TB IgA EIA (39)	0.86	(0.67 - 0.96)
TB IgA EIA (46)	0.48	(0.30 - 0.67)
TB IgA EIA (50)	0.47	(0.30 - 0.65)
TB IgG EIA (46)	0.77	(0.59 - 0.90)
TBGL (47)	0.72	(0.58 - 0.84)
TBGL (54)	0.85	(0.79 - 0.90)
TBGL (56)	0.89	(0.82 - 0.94)
