# Supplementary material for: Commercial Serological Antibody Detection Tests for the Diagnosis of Pulmonary Tuberculosis: A Systematic Review
Source: PLoS Med. 2007 Jun 12;4(6):e202. doi: 10.1371/journal.pmed.0040202 (PMC1891320; doi:10.1371/journal.pmed.0040202)
Supplement: Supplementary file 3 [file pmed.0040202.sg003.pdf]

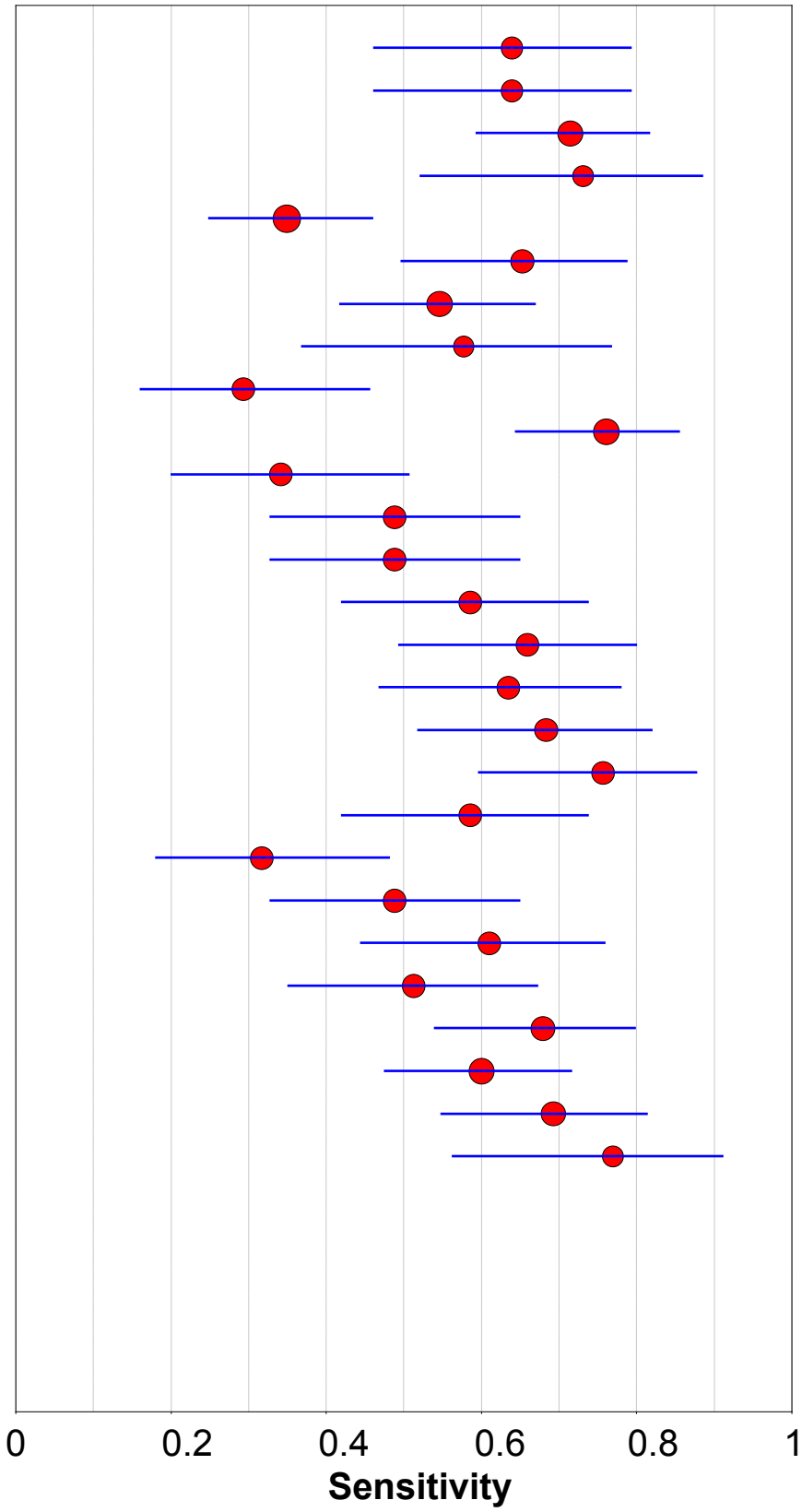

	Sensitivity (95% CI)	
Anda IgA (41)	0.64	(0.46 - 0.79)
Anda IgG (41)	0.64	(0.46 - 0.79)
Anda IgG (52)	0.71	(0.59 - 0.82)
Anda IgG (56)	0.73	(0.52 - 0.88)
Anda IgG (64)	0.35	(0.25 - 0.46)
Detect TB (42)	0.65	(0.50 - 0.79)
KAT (45)	0.55	(0.42 - 0.67)
MycoDot (56)	0.58	(0.37 - 0.77)
P Plus (48)	0.29	(0.16 - 0.46)
P Plus (63)	0.76	(0.64 - 0.85)
Path Myco A (48)	0.34	(0.20 - 0.51)
Path Myco A/P Plus (48)	0.49	(0.33 - 0.65)
Path Myco G (48)	0.49	(0.33 - 0.65)
Path Myco G/A (48)	0.59	(0.42 - 0.74)
Path Myco G/A/P Plus (48)	0.66	(0.49 - 0.80)
Path Myco G/M (48)	0.63	(0.47 - 0.78)
Path Myco G/M/A (48)	0.68	(0.52 - 0.82)
Path Myco G/M/A/P Plus (48)	0.76	(0.60 - 0.88)
Path Myco G/P Plus (48)	0.59	(0.42 - 0.74)
Path Myco M (48)	0.32	(0.18 - 0.48)
Path Myco M/A (48)	0.49	(0.33 - 0.65)
Path Myco M/A/P Plus (48)	0.61	(0.45 - 0.76)
Path Myco M/P Plus (48)	0.51	(0.35 - 0.67)
TB IgA EIA (39)	0.68	(0.54 - 0.80)
TBGL (53)	0.60	(0.48 - 0.72)
TBGL (54)	0.69	(0.55 - 0.81)
TBGL (56)	0.77	(0.56 - 0.91)
