# Supplementary material for: Commercial Serological Antibody Detection Tests for the Diagnosis of Pulmonary Tuberculosis: A Systematic Review
Source: PLoS Med. 2007 Jun 12;4(6):e202. doi: 10.1371/journal.pmed.0040202 (PMC1891320; doi:10.1371/journal.pmed.0040202)
Supplement: Supplementary file 4 [file pmed.0040202.sg004.pdf]

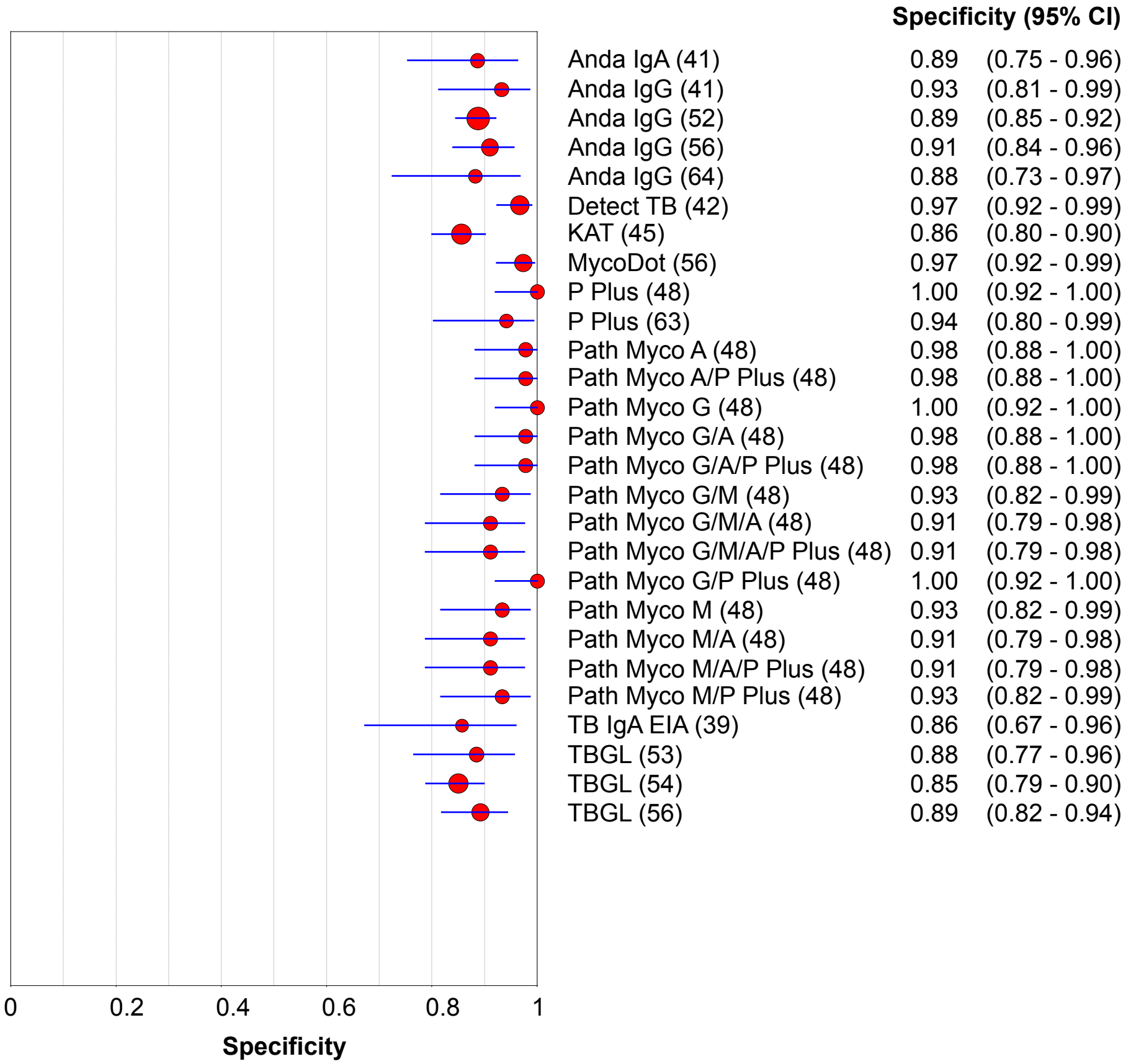
Specificity (95% CI)
Anda IgA (41) 0.89 (0.75 - 0.96)
Anda IgG (41) 0.93 (0.81 - 0.99)
Anda IgG (52) 0.89 (0.85 - 0.92)
Anda IgG (56) 0.91 (0.84 - 0.96)
Anda IgG (64) 0.88 (0.73 - 0.97)
Detect TB (42) 0.97 (0.92 - 0.99)
KAT (45) 0.86 (0.80 - 0.90)
MycoDot (56) 0.97 (0.92 - 0.99)
P Plus (48) 1.00 (0.92 - 1.00)
P Plus (63) 0.94 (0.80 - 0.99)
Path Myco A (48) 0.98 (0.88 - 1.00)
Path Myco A/P Plus (48) 0.98 (0.88 - 1.00)
Path Myco G (48) 1.00 (0.92 - 1.00)
Path Myco G/A (48) 0.98 (0.88 - 1.00)
Path Myco G/A/P Plus (48) 0.98 (0.88 - 1.00)
Path Myco G/M (48) 0.93 (0.82 - 0.99)
Path Myco G/M/A (48) 0.91 (0.79 - 0.98)
Path Myco G/M/A/P Plus (48) 0.91 (0.79 - 0.98)
Path Myco G/P Plus (48) 1.00 (0.92 - 1.00)
Path Myco M (48) 0.93 (0.82 - 0.99)
Path Myco M/A (48) 0.91 (0.79 - 0.98)
Path Myco M/A/P Plus (48) 0.91 (0.79 - 0.98)
Path Myco M/P Plus (48) 0.93 (0.82 - 0.99)
TB IgA EIA (39) 0.86 (0.67 - 0.96)
TBGL (53) 0.88 (0.77 - 0.96)
TBGL (54) 0.85 (0.79 - 0.90)
TBGL (56) 0.89 (0.82 - 0.94)
0
0.2
0.4
0.6
0.8
1
Specificity
