# Supplementary material for: Commercial Serological Antibody Detection Tests for the Diagnosis of Pulmonary Tuberculosis: A Systematic Review
Source: PLoS Med. 2007 Jun 12;4(6):e202. doi: 10.1371/journal.pmed.0040202 (PMC1891320; doi:10.1371/journal.pmed.0040202)
Supplement: Supplementary file 5 [file pmed.0040202.sg005.pdf]

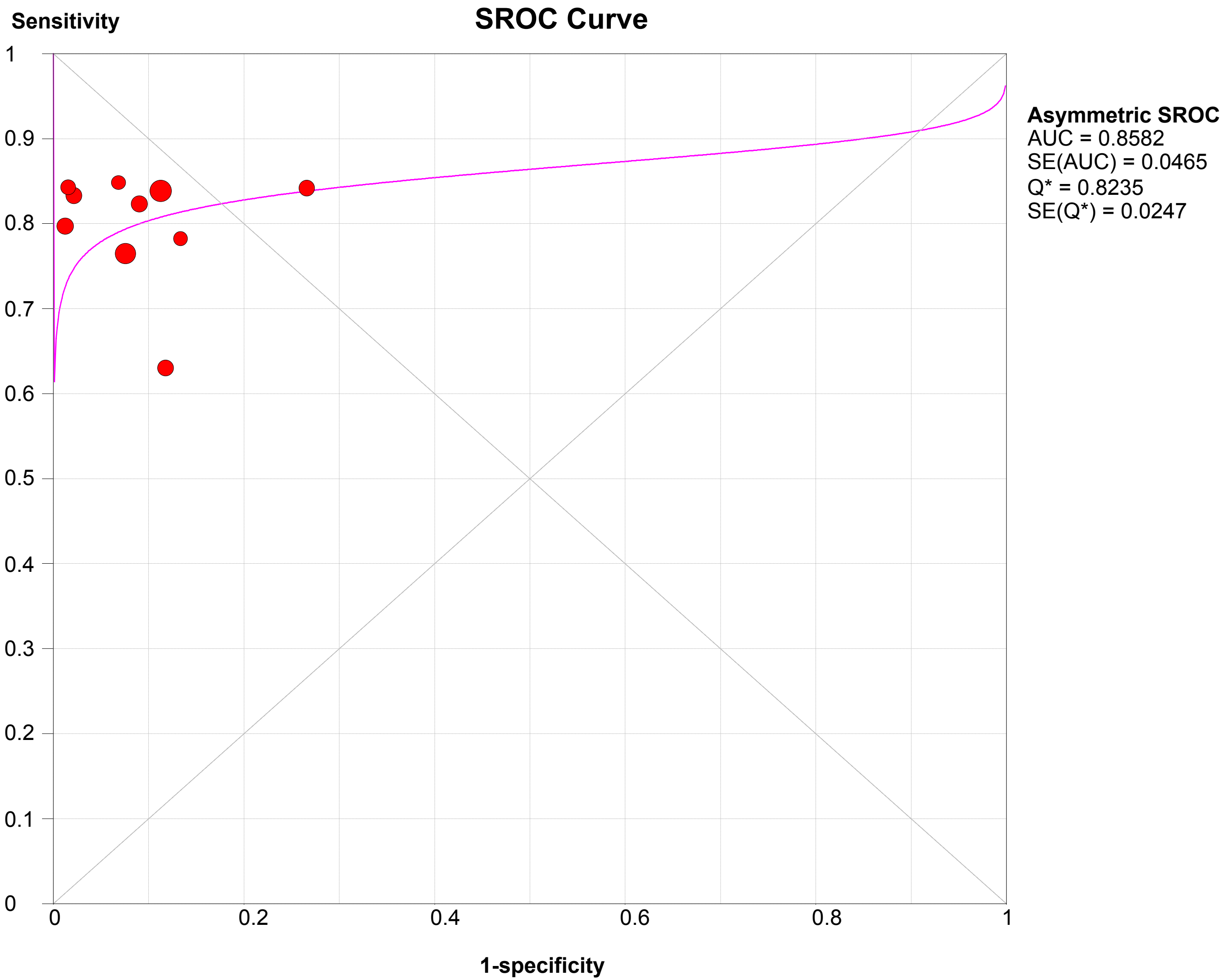

SROC Curve
Sensitivity
1
0.9
0.8
0.7
0.6
0.5
0.4
0.3
0.2
0.1
0
0
0.2
0.4
0.6
0.8
1
1-specificity
Asymmetric SROC
AUC = 0.8582
SE(AUC) = 0.0465
Q* = 0.8235
SE(Q*) = 0.0247
