# Supplementary material for: Commercial Serological Antibody Detection Tests for the Diagnosis of Pulmonary Tuberculosis: A Systematic Review
Source: PLoS Med. 2007 Jun 12;4(6):e202. doi: 10.1371/journal.pmed.0040202 (PMC1891320; doi:10.1371/journal.pmed.0040202)
Supplement: Supplementary file 6 [file pmed.0040202.sg006.pdf]

A

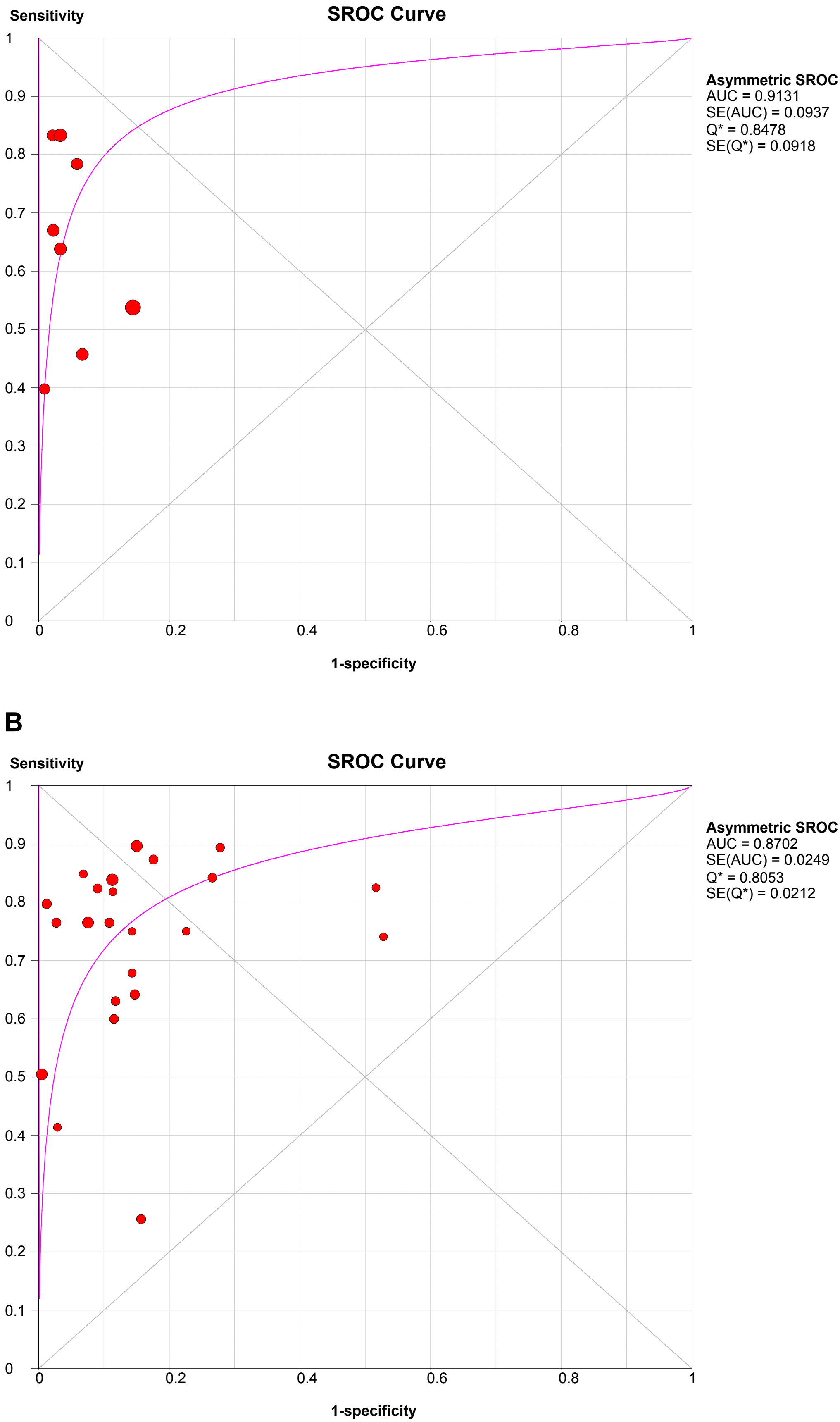
Sensitivity
SROC Curve
1
0.9
0.8
0.7
0.6
0.5
0.4
0.3
0.2
0.1
0
0
0.2
0.4
0.6
0.8
1
1-specificity
Asymmetric SROC
AUC = 0.9131
SE(AUC) = 0.0937
Q* = 0.8478
SE(Q*) = 0.0918
B
Sensitivity
SROC Curve
1
0.9
0.8
0.7
0.6
0.5
0.4
0.3
0.2
0.1
0
0
0.2
0.4
0.6
0.8
1
1-specificity
Asymmetric SROC
AUC = 0.8702
SE(AUC) = 0.0249
Q* = 0.8053
SE(Q*) = 0.0212
